# Short Implants versus Standard Implants and Sinus Floor Elevation in Atrophic Posterior Maxilla: A Systematic Review and Meta-Analysis of Randomized Clinical Trials with ≥5 Years’ Follow-Up

**DOI:** 10.3390/jpm13020169

**Published:** 2023-01-18

**Authors:** Alexandru Mester, Florin Onisor, Dario Di Stasio, Andra Piciu, Adriana-Maria Cosma, Simion Bran

**Affiliations:** 1 Department of Oral Health, University of Medicine and Pharmacy “Iuliu Hatieganu”, 400006 Cluj-Napoca, Romania; 2 Department of Maxillofacial Surgery and Implantology, University of Medicine and Pharmacy “Iuliu Hatieganu”, 400012 Cluj-Napoca, Romania; 3 Multidisciplinary Department of Medical-Surgical and Dental Specialties, University of Campania “Luigi Vanvitelli”, 80138 Naples, Italy; 4 Department of Medical Oncology, University of Medicine and Pharmacy “Iuliu Hatieganu”, 400012 Cluj-Napoca, Romania; 5 Faculty of Dental Medicine, University of Medicine and Pharmacy “Iuliu Hatieganu”, 400006 Cluj-Napoca, Romania

**Keywords:** short implant, ultrashort implant, sinus lift, sinus floor elevation

## Abstract

*Background and objectives*: The aim of this systematic review with meta-analysis was to assess the performance of short implants in comparison with standard implants and sinus floor elevation in atrophic posterior maxilla. *Materials and methods*: The protocol of the study was registered in the PROSPERO database (CRD42022375320). An electronic search on three databases (PubMed, Scopus, Web of Science) was performed to find randomized clinical trials (RCTs) with ≥5 years’ follow-up, published until December 2022. Risk of bias (ROB) was calculated using Cochrane ROB. A meta-analysis was performed for primary (implant survival rate, ISR) and secondary outcomes (marginal bone loss, MBL; biological and prosthetic complications). *Results*: Of 1619 articles, 5 RCTs met the inclusion criteria. The ISR showed a risk ratio (RR) of 0.97 [0.94, 1.00] (CI 95%), *p* = 0.07. The MBL indicated a WMD of −0.29 [−0.49, −0.09] (CI 95%), *p* = 0.005. Biological complications showed a RR of 0.46 [0.23, 0.91] (CI 95%), *p* = 0.03. Prosthetic complications showed a RR of 1.51 [0.64, 3.55] (CI 95%), *p* = 0.34. *Conclusions*: The available evidence suggests that short implants might be used as an alternative to standard implants and sinus floor elevation. After 5 years, in terms of ISR, standard implants and sinus floor elevation showed a higher survival rate comparted to short implants, although statistical significance was not achieved. Future RCTs with long-term follow-up are needed to draw a clear conclusion on the advantages of one method over another.

## 1. Introduction

Atrophic posterior maxilla still represents a challenging situation in implant dentistry. This fact is determined by the resorption of alveolar ridge and maxillary sinus pneumatization [1]. In order to rehabilitate the maxillary edentulous ridge, a variety of prosthetic solutions and surgical techniques have been proposed [1,2]. The removable prosthesis is a common and successful option in rehabilitation of this area with long-term successful outcomes [3]. As with any treatment, the removable prosthesis has several disadvantages, such as being ill-fitting, the adjustment period, loss of retention, ulceration, plaque accumulation, or fracture [3].

To combat the disadvantages of removable prostheses, a variety of surgical techniques have been proposed to reconstruct resorbed posterior maxilla, and then, to use implant-supported fixed prostheses [2]. These techniques are maxillary sinus floor elevation combined with bone grafts, guided bone regeneration (GBR), onlay/inter-positional grafts, short implants, tilted implants, and zygomatic implants [2,4]. Of all these techniques, the maxillary sinus floor elevation is one of the most frequently used techniques in current clinical practice [5]. Sinus lift can be done via a trans-alveolar ridge or via the lateral window technique using different types of bone grafts (autogenous bone, bone substitutes or a mixture of both) in order to maintain space and determine bone regeneration [2,5]. Being a sensitive technique, with this procedure, clinicians may encounter several complications, such as perforation of the Schneiderian membrane, sinusitis, graft failure, post-operative pain, bleeding, or even migration of dental implants in the maxillary sinus [6,7].

Therefore, the use of short implants has been introduced in order to reduce the complications of sinus floor elevation. The definition of a short implant is considered to be one with a length under 8 mm [8]. The use of such implants has the advantages of eliminating the elevation of the maxillary sinus floor, and reducing post-operative complications, treatment time, and cost [8]. However, several studies have mentioned failure of short implants in comparison to standard implants, such as early implant failure, and characteristics of the implant surface [9,10,11,12].

From the current literature, there is no consensus on which therapy is better for atrophic posterior maxilla. The aim of our systematic review with meta-analysis was to assess the clinical performance of short implants in comparison with standard implants and sinus floor elevation in atrophic posterior maxilla.

## 2. Materials and Methods

### 2.1. Protocol and Registration of the Study

This systematic analysis was designed according to the PRISMA guidelines [13]. A priori, the protocol details of this review were submitted and registered in the PROSPERO database (code number CRD42022375320).

### 2.2. Participants, Intervention, Comparison, Outcome (PICO) Question

The question of focus was elaborated according to the PICO question: “In patients with atrophic posterior maxilla (P), what is the ≥5 years’ efficiency of short implants (I) in comparison with sinus floor elevation and standard implants (C) in terms of implant survival rate; marginal bone loss (MBL), complications (O)?”

PICO elements were as follows:

- Participants: healthy systemic patients, ≥18 years, with atrophic posterior maxilla in need of implant placement;
- Intervention: ultra-short/short implants with a length ≤7 mm;
- Comparison: sinus floor elevation and standard implants with a length ≥8 mm;
- Outcome: implant survival rate (primary outcome); MBL, biological complications (i.e., peri-implant mucositis, peri-implantitis), prosthetic complications (i.e., implant supported prosthetic fracture, screw, abutment fracture/loosening, implant fracture) (secondary outcome);
- Study type: randomized clinical trials (RCTs) or prospective controlled clinical trials (CCTs) with a follow-up ≥5 years.

### 2.3. Inclusion and Exclusion Criteria for RCTs

The inclusion criteria were:

- Randomized clinical trials (RCTs) or controlled clinical trials (CCTs);
- Comparison short implant (≤7 mm) and standard implants (≥8 mm) and sinus floor elevation in the same RCT;
- RCT with a follow-up ≥5 years;
- Implants restored with fixed partial dentures.

The exclusion criteria were:

- In vitro, animal studies; no clinical trials, cross-sectional, cohort studies; systematic or narrative reviews, case reports, case series, monographs, letters to the editor;
- RCT with insufficient, missing or unpublished data;
- RCT with a follow-up of <5 years;
- Articles published in another language than English.

### 2.4. Search Methods

An electronic search was performed on 4 December 2022 by two independent reviewers (A.M. and F.O.) in the PubMed, Web of Science and Scopus database and included articles published until December 2022. A grey literature search in the OpenGrey and ClinicalTrial.gov database was done. A manual search in journals specialized in implantology was carried out (Journal of Periodontal Research, Journal of Periodontology, Journal of Clinical Periodontology, European Journal of Oral Sciences, European Journal of Oral Implantology, Dental Journal, British Journal of Oral and Maxillofacial Surgery, Clinical Implant Dentistry and Related Research, Clinical Oral Implants Research, Clinical Oral Investigations, Implant Dentistry, International Journal of Oral and Maxillofacial Implants, International Journal of Oral and Maxillofacial Surgery, International Journal of Periodontics and Restorative Dentistry, Journal of Dental Research, Journal of Dentistry, Journal of Implantology, Journal of Maxillofacial and Oral Surgery, Journal of Oral and Maxillofacial Surgery and Oral Surgery, Oral Medicine, Oral Pathology, Oral Radiology, Journal of Indian Society of Periodontology).

To identify relevant articles, the following search strategy was applied: (‘short implant’ OR ‘ultrashort implant’ OR ‘standard implant’ OR ‘dental implant’) AND (‘sinus lift’ OR ‘sinus floor augmentation’ OR ‘sinus floor elevation’ OR ‘sinus membrane elevation’ OR ‘lateral approach sinus floor elevation’ OR ‘osteotome sinus floor elevation’ OR ‘atrophic posterior maxilla’ OR ‘edentulous posterior maxilla’). Firstly, titles and abstracts from the electronic searches were screened and irrelevant articles were excluded. Secondly, after removing the duplicates, full-text articles previously obtained were examined and those who met to the inclusion criteria were downloaded. If any disagreements related to the selection of the studies was noted, a third reviewer (S.B.) intervened with an additional resolution.

### 2.5. Data Extraction

The following data from the included studies were taken: first author, year of study, country, reference, type of RCT, patients characteristics and implants, implant treatment modality, type of sinus lift surgery, type of prosthetic restoration, primary outcome, secondary outcomes, and conclusions.

### 2.6. Risk of Bias

The risk of bias was quantified using the Cochrane Risk of Bias tool version 2.0 [14]. For each RCT included, seven domains were assessed (random sequence generation; allocation concealment; blinding of participants and/or personnel involved in the study; blinding of outcome assessment; incomplete outcome data reporting; selective reporting of outcomes; other sources of bias). Each domain was analyzed by two independent reviewers (A.M., F.O.) and a third reviewer (S.B.) intervened if any disagreement was present. These domains received a quality grade (low, unclear, or high).

### 2.7. Statistical Analysis

A meta-analysis was performed using RevMan version 5.4 from the Cochrane Collaboration 2020 [15]. A random effect model with a confidence interval (CI) of 95% was used. For the implant survival rate (primary outcome), biological and prosthetic complications (secondary outcomes), risk ratio (RR) (CI 95%) was assessed using a chi-square test [Mantel–Haenszel (M–H)]. Due to the clinical heterogeneity detected between studies, a random-effects model was applied, in order to analyze effect sizes. For MBL (secondary outcome), a weighted mean difference (WMD) (CI 95%) with sample size, inverse variance (IV), and standard error was calculated. The value *p* < 0.05 was considered statistically significant. The heterogeneity among the studies was evaluated with an I-squared statistic test (*I*^2^). I^2^ values lower than 30% indicated low heterogeneity, values between 30–60% indicated moderate heterogeneity, and values over 60% indicated substantial heterogeneity.

## 3. Results

### 3.1. Study Selection

**Figure 1:**
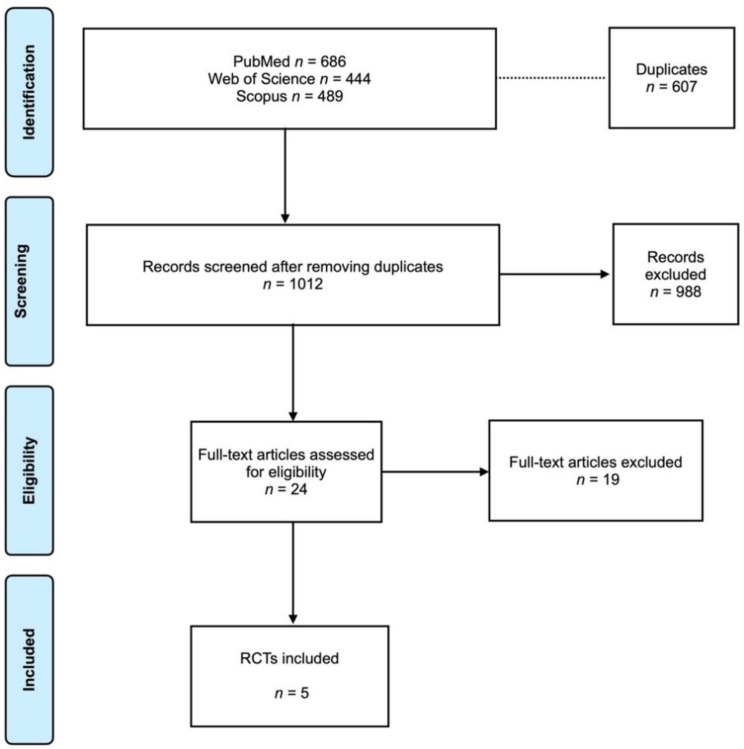
Prisma flowchart.

Search results are presented in a Prisma flowchart (Figure 1). The electronic search on the selected database determined a total of 1619 articles. The grey literature was also assessed with no articles to correspond. After removing the duplicates (607 articles), 1012 articles were screened. Of these, 24 articles were full-text assessed for eligibility according to the inclusion criteria. After the evaluation, 19 articles were excluded (reason for exclusion is presented in the Supplementary File, Table S1. In the end, 5 RCTs [16,17,18,19,20] were included in the analysis. The coefficient Cohen’s kappa for inter-reviewer agreement was 0.96.

### 3.2. Description of the Included RCTs

The RCTs were published between 2018 and 2019 and conducted in Switzerland, Sweden, Italy and the Netherlands. The study design consisted of an open-prospective RCT multicenter, two-arms parallel group RCT multicenter and split-mouth RCT (Table 1). The total number of patients was 203, of which 84 patients were treated with short implants (S.I.) and 84 with standard implants + sinus lift elevation (Std. I. + S.L.); in regards to the discrepancy of patients’ number, 2 RCTs [18,19] did not report how many patients were in each type of treatment. The total number of inserted implants was 393, of which, 190 were short implants and 203 were standard implants. The length and diameter of short implants was 5–6 mm and 4–6 mm, respectively. The length and diameter of standard implants was 10–15 mm and 4–5 mm, respectively. All RCTs for sinus floor elevation used a lateral window technique using a bone graft with a resorbable collagen membrane [16,17,18,19] or only a bone graft plus an autogenous bone [20]. The majority of the RCTs used cemented crowns as final prosthetics, and only one RCT used also screw-retained crowns.

### 3.3. Risk of Bias Assessment

The results of the Cochrane ROB assessment are presented in Table 2. Most RCTs were considered to have a high ROB.

### 3.4. Statistical Analysis of Primary and Secondary Outcomes

**Figure 2:**
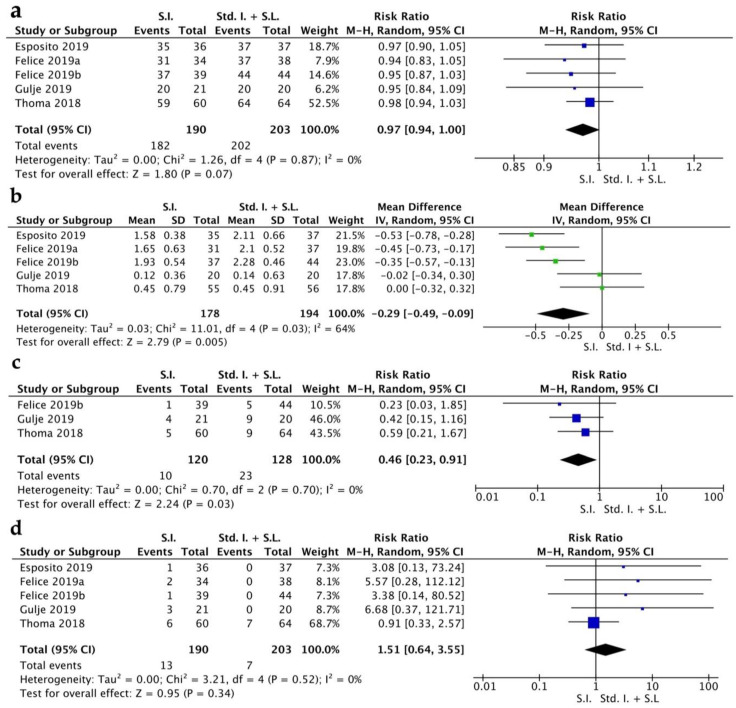
Forest plot for implant survival rate (**a**), MBL (**b**), biological complications (**c**), prosthetic complications (**d**). Comparison of short implants (S.I.) versus standard implants and sinus lift (Std. I. + S.L.) [16,17,18,19,20].

The implant survival rate (ISR) had a RR of 0.97 [0.94, 1.00] (CI 95%). Heterogeneity was low (I^2^ = 0%) and the random effect model was *p* = 0.07 (Figure 2a). The MBL indicated a WMD of −0.29 [−0.49, −0.09] (CI 95%) with a high grade of heterogeneity (I^2^ = 64%) and statistical significance was achieved *p* = 0.005 (Figure 2b). Biological complications were quantified from three RCTs and showed a RR of 0.46 [0.23, 0.91] (CI 95%), with a low grade of heterogeneity (I^2^ = 0%) and a random effect model of *p* = 0.03 (Figure 2c). Prosthetic complications showed a RR of 1.51 [0.64, 3.55] (CI 95%), with a low grade of heterogeneity (I^2^ = 0%) and a random effect model of *p* = 0.34 (Figure 2d).

## 4. Discussion

The aim of this systematic review with meta-analysis was to compare the results of short implants and standard implants plus sinus floor elevation in atrophic posterior maxilla in terms of ISR, MBL, biological and prosthetic complications.

The results of ISR indicated no statistical difference between the two therapies; however, survival rate was 95.78% for short implants and 99.5% standard implants during the 5-year follow-up. Guida and coworkers [21] obtained the ISR of 95.40% for short implants and 98.44% for standard implants at 5 years’ follow-up. The authors mentioned that no statistical significance was achieved with a RR of 0.98 [95% CI: (0.94, 1.01); *p* = 0.21]. In the analysis, the authors included implants inserted in the mandibular region. In the meta-analysis of Toledano and coworkers [22], the authors included RCTs with a follow-up longer than 1 year with a RR value of 1.02 [95% CI: (1.00, 1.05); *p* = 0.09], suggesting that the ISR was similar for both types of implants. The meta-analysis of Bitinas et al. [9] included 3 RCTs with a follow-up of 5 years and also showed a statistically insignificant difference with a RR value of 0.03 [95% CI: −0.07 to 0.13, (*p* = 0.52)]. The meta-analysis of Lozano-Carrascal et al. [23] included RCTs with a follow-up longer than 3 years and obtained no statistical significance with a RR value of 1.08 [95% CI: (0.42, 2.83); *p* = 0.8). Iezzi and coworkers stated in their meta-analysis that high ISR was obtained in short implants in comparison with standard implants [24]. As seen, different results may be found in the electronic literature. Other RCTs have indicated that the ISR for both implants are the same [25,26]. These inconsistencies around systematic reviews may be due to the study population and type of implant system used [22]. However, standard implants with sinus lifts showed better outcomes, and the sinus lift procedure still remains a sensitive technique. Several studies have indicated that this is a safe and predictable technique regardless of the biomaterial used, in terms of clinical and histopathological assessment, and also in patient-reported outcome measurements [27,28].

The MBL indicated a statistical significance between the two therapies. Guida et al. [21] reported a higher MBL at 5 years for standard implants comparted to short implants [0.6 mm (95% CI: 0.42, 0.78; *p* < 0.00001)]. Toledano et al. reported the MBL of 0.23 mm [95% CI: (0.07, 0.39); *p* = 0.005], indicating that the MBL was higher for standard implants [22]. Bitinas et al. [9] obtained a MBL of −0.45 mm [95% CI: (−0.87, −0.02); *p* = 0.04]. Lozano-Carrascal et al. [23] indicated that MBL was in favor of short implants. Iezzi et al. [24] also indicated a significantly lower MBL associated with short implants compared to standard implants. MBL might be influenced by several factors, which includes the type of implant (design, surface configuration), surgical preparation, and surgeon experience [21,22].

The biological complications indicated a statistical significance between the two therapies. Guida et al. [21] reported no statistical significance with a RR value of 1.02 [95% CI: (0.30, 3.47); *p* = 0.98). Lozano-Carrascal et al. [23] showed a RR of 0.46 [95% CI: (0.22, 0.95); *p* = 0.037]. As seen, there are not many systematic reviews which report on biological complications. In addition, from our 5 RCTs included, only 3 RCTs mentioned biological complications (peri-implant mucositis or peri-implantitis). Another difficulty seen in the RCTs included was in regard to the diagnosis of peri-implant disease.

The prosthetic complications indicated no statistical differences. Guida et al. [21] also found no statistical significance at 0.80 (95% CI: 0.47, 1.34; *p* = 0.39). Lozano-Carrascal et al. [23] indicated a RR of 1.52 [95% CI: (0.91, 2.54); *p* = 1], favoring the standard implants. The variation of the results might have been due to several factors, such as the type of edentulism, prosthetic loading, or type of implant-supported prosthetic (fixed/cemented). Guida and coworkers addressed the issue of other important factors, such as bruxism, smoking, bone quality or implant stability [21].

This systematic review with meta-analysis had several limitations due to heterogeneity and a lack of available information. The main limitation was the low number of the available RCTs with >5 years’ follow-up. Secondly, incomplete information has been reported about biological complications (e.g., peri-implant mucositis, peri-implantitis). In regard to confounding factors, no subgroup analysis could be assessed. Furthermore, in the included RCTs, there was insufficient information in regard to the type of implant-supported reconstruction.

## 5. Conclusions

The available evidence of this present study suggests that short implants might be used as an alternative to standard implants and sinus floor elevation. After 5 years, in terms of implant survival rate, standard implants and sinus floor elevation showed a higher survival rate compared to short implants; however, statistical significance could not be achieved. Secondary outcomes indicated statistical significance in marginal bone loss and biological complications, and there was no statistical significance in prosthetic complications. Future RCTs with long-term follow-up are needed to draw a clear conclusion on the advantages of one method over another.

## Figures and Tables

**Table 1:** Characteristics of the included RCTs.

Author. Year. Country. Reference	Study Design	Patients	Surgical Treatment Modality	Type of Implant	Prosthetic Type	Implant Survival Rate	Marginal Bone Loss	Complications	Conclusions
Thoma. 2018. Switzerland [16]	Open Prospective RCT multicenter	*n* = 90 S.I.: *n* = 44 Std. I. + S.L.: *n* = 46	S.I.: implant insertion Std. I. + S.L.: sinus floor elevation, lateral window technique, bone graft, resorbable collagen membrane	S.I.: *n* = 60 (titanium implant: length 6 mm, diameter 4 mm) Std. I. + S.L.: *n* = 64 (titanium implant: length 11–15 mm, diameter 4 mm)	Non-splinted single crown screw-retained/cemented	S.I.: 98.5% (1 implant) Std. I. + S.L.: 100%	S.I.: 0.54 mm ± 0.87 Std. I. + S.L.: 0.46 mm ± 1.00	S.I.: Biological: *n* = 5 Prosthetic: *n* = 21 Std. I. + S.L. Biological: *n* = 9 Prosthetic: *n* = 15	Both treatments were suitable for atrophic posterior maxilla.
Esposito. 2019. Sweden [17]	Two arms parallel group RCT multicenter	*n* = 40 S.I.: *n* = 20 Std. I. + S.L.: *n* = 20	S.I.: implant insertion Std. I. + S.L.: sinus floor elevation, lateral window technique, bone graft, resorbable collagen membrane	S.I.: *n* = 36 (titanium implant: length 5 mm, diameter 5 mm) Std. I. + S.L.: *n* = 37 (titanium implant: length 10–15 mm, diameter 5 mm)	Screw-retained/cemented	S.I.: 97.2% (1 implant) Std. I. + S.L.: 100%	S.I.: 1.58 mm ± 0.38 Std. I. + S.L.: 2.11 mm ± 0.66	S.I.: Biological: NA Prosthetic: *n* = 1 Std. I. + S.L. Biological: NA Prosthetic: *n* = 0	S.I. achieved similar results with Std. I. + S.L.
Felice. 2019. Italy [18]	Split mouth RCT	*n* = 15 S.I.: *n* = NA Std. I. + S.L.: *n* = NA	S.I.: implant insertion Std. I. + S.L.: sinus floor elevation, lateral window technique, bone graft, resorbable collagen membrane	S.I.: *n* = 34 (titanium implant: length 5 mm, diameter 6 mm) Std. I. + S.L.: *n* = 38 (titanium implant: length ≤10 mm, diameter 4 mm)	Cemented	S.I.: 91.2% (3 implants) Std. I. + S.L.: 97.4% (1 implant)	S.I.: 1.65 mm ± 0.63 Std. I. + S.L.: 2.10 mm ± 0.52	S.I.: Biological: NA Prosthetic: *n* = 2 Std. I. + S.L. Biological: NA Prosthetic: *n* = 0	S.I. achieved similar results with Std. I. + S.L.
Felice. 2019. Italy [19]	Split mouth RCT	*n* = 20 S.I.: *n* = NA Std. I. + S.L.: *n* = NA	S.I.: implant insertion Std. I. + S.L.: sinus floor elevation, lateral window technique, bone graft, resorbable collagen membrane	S.I.: *n* = 39 (titanium implant: length 6 mm, diameter 4 mm) Std. I. + S.L.: *n* = 44 (titanium implant: length 10–15 mm, diameter 4 mm)	Cemented	S.I.: 95.5% (2 implants) Std. I. + S.L.: 100%	S.I.: 1.93 mm ± 0.54 Std. I. + S.L.: 2.28 mm ± 0.46	S.I.: Biological: *n* = 1 Prosthetic: *n* = 1 Std. I. + S.L. Biological: *n* = 5 Prosthetic: *n* = 0	S.I. achieved similar results with Std. I. + S.L.
Gulje. 2019. The Netherlands [20]	RCT	*n* = 38 S.I.: *n* = 20 Std. I. + S.L.: *n* = 18	S.I.: implant insertion Std. I. + S.L.: sinus floor elevation, lateral window technique, bone graft	S.I.: *n* = 21 (titanium implant: length 6 mm, diameter 4 mm) Std. I. + S.L.: *n* = 20 (titanium implant: length 11 mm, diameter 4 mm)	Cemented	S.I.: 94.7% (1 implants) Std. I. + S.L.: 100%	S.I.: 0.12 mm ± 0.36 Std. I. + S.L.: 0.14 mm ± 0.63	S.I.: Biological: *n* = 4 Prosthetic: *n* = 3 Std. I. + S.L. Biological: *n* = 9 Prosthetic: *n* = 0	Std. I. + S.L. are equally successful compared to S.I.

NA: not available; S.I.: short implants; Std. I. + S.L.: standard implants + sinus lift.

**Table 2:** Cochrane ROB assessment.

Random sequence generation	Low	Low	Low	High	Low
Allocation concealment	Low	Low	Low	Low	Low
Blinding of the participants and personnel	Unclear	High	High	High	High
Blinding of outcome assessment	Low	Low	Low	High	Unclear
Incomplete outcome data	Unclear	Unclear	Unclear	Low	Unclear
Selective reporting	Low	Low	Low	Low	Low
Other bias	Low	Low	Low	Low	Low
Estimated ROB	Unclear	High	High	High	
Study. Year. Reference	Esposito 2019 [17]	Felice 2019 [18]	Felice 2019 [19]	Gulje 2019 [20]	Thoma 2018 [16]

## Data Availability

Not applicable.

## Supplementary Materials

The following supporting information can be downloaded at: https://www.mdpi.com/article/10.3390/jpm13020169/s1, Table S1: Reason for article exclusion [27,29,30,31,32,33,34,35,36,37,38,39,40,41,42,43,44,45,46].
