# Supplementary material for: Short Implants versus Standard Implants and Sinus Floor Elevation in Atrophic Posterior Maxilla: A Systematic Review and Meta-Analysis of Randomized Clinical Trials with ≥5 Years’ Follow-Up
Source: J Pers Med. 2023 Jan 18;13(2):169. doi: 10.3390/jpm13020169 (PMC9960634; doi:10.3390/jpm13020169)
Supplement: Supplementary file 1 [file jpm-13-00169-s001.zip › jpm-2122103-supplementary.pdf]

Table S1. Reason for article exclusion

Reference	Reason for exclusion
Dasmah et al [29]	Prospective study
Cannizzaro et al [30]	Lack of using only short implants in the comparison group
Romeo et al [31]	Treatment in mandibular arch
Rossi et al [32]	Lack of using standard implants and sinus floor elevation in the comparison group
Shi et al [33]	No data available
Nedir et al [34]	Lack of using only short implants without sinus floor elevation in the comparison group
Toljanic et al [35]	No use of short implants
Gherlone et al [36]	Prospective longitudinal study
Cannizzaro et al [37]	Lack comparison short implant (≤6 mm) and standard implants (≥ 8 mm) and sinus floor elevation in the same study
Storelli et al [38]	Lack of using standard implants and sinus floor elevation in the comparison group
Naenni et al [39]	Lack of using standard implants and sinus floor elevation in the comparison group
Meloni et al [40]	Lack of using only short implants in the comparison group
Testori et al [41]	Prospective study
Slot et al [42]	All-on- six concept
Qian et al [43]	Lack of using only short implants in the comparison group
Velasco-Ortega et al [27]	Lack of using only short implants in the comparison group
Gulje et al [44]	Short implant in mandibular posterior region
Zadeh et al [45]	Follow-up 3 years
Barausse et al [46]	Follow-up 3 years

References

27. Velasco-Ortega, E.; Valente, N.A.; Iezzi, G.; Petrini, M.; Derchi, G.; Barone, A. Maxillary sinus augmentation with three different biomaterials: Histological, histomorphometric, clinical, and patient-reported outcomes from a randomized controlled trial. Clin. Implant Dent. Relat. Res. 2021, 23, 86–95.
29. Dasmah, A.; Thor, A.; Ekestubbe, A.; Sennerby, L.; Rasmusson, L. Marginal bone-level alterations at implants installed in block versus particulate onlay bone grafts mixed with platelet-rich plasma in atrophic maxilla. a prospective 5-year follow-up study of 15 patients. Clin. Implant Dent. Relat. Res. 2013, 15, 7–14.
30. Cannizzaro, G.; Felice, P.; Minciarelli, A.F.; Leone, M.; Viola, P.; Esposito, M. Early implant loading in the atrophic posterior maxilla: 1-stage lateral versus crestal sinus lift and 8 mm hydroxyapatite-coated implants. A 5-year randomised controlled trial. *Eur. J. Oral Implantol.* **2013**, *6*, 13–25.
31. Romeo, E.; Storelli, S.; Casano, G.; Scanferla, M.; Botticelli, D. Six-mm versus 10-mm long implants in the rehabilitation of posterior edentulous jaws: a 5-year follow-up of a randomised controlled trial. *Eur. J. Oral Implantol.* **2014**, *7*, 371–381.
32. Rossi, F.; Botticelli, D.; Cesaretti, G.; De Santis, E.; Storelli, S.; Lang, N.P. Use of short implants (6 mm) in a single-tooth replacement: a 5-year follow-up prospective randomized controlled multicenter clinical study. *Clin. Oral Implants Res.* **2016**, *27*, 458–464.
33. Shi, J.-Y.; Gu, Y.-X.; Qiao, S.-C.; Zhuang, L.-F.; Zhang, X.-M.; Lai, H.-C. Clinical evaluation of short 6-mm implants alone, short 8-mm implants combined with osteotome sinus floor elevation and standard 10-mm implants combined with osteotome sinus floor elevation in posterior maxillae: study protocol for a randomized controlle. *Trials* **2015**, *16*, 324.
34. Nedir, R.; Nurdin, N.; Abi Najm, S.; El Hage, M.; Bischof, M. Short implants placed with or without grafting into atrophic sinuses: the 5-year results of a prospective randomized controlled study. *Clin. Oral Implants Res.* **2017**, *28*, 877–886.
35. Toljanic, J.A.; Ekstrand, K.; Baer, R.A.; Thor, A. Immediate Loading of Implants in the Edentulous Maxilla with a Fixed Provisional Restoration without Bone Augmentation: A Report on 5-Year

Outcomes Data Obtained from a Prospective Clinical Trial. *Int. J. Oral Maxillofac. Implants* **2016**, *31*, 1164–1170.

36. Gherlone, E.F.; Sannino, G.; Rapanelli, A.; Crespi, R.; Gastaldi, G.; Capparé, P. Prefabricated Bar System for Immediate Loading in Edentulous Patients: A 5-Year Follow-Up Prospective Longitudinal Study. *Biomed Res. Int.* **2018**, *2018*, 7352125.
37. Cannizzaro, G.; Felice, P.; Ippolito, D.R.; Velasco-Ortega, E.; Esposito, M. Immediate loading of fixed cross-arch prostheses supported by flapless-placed 5 mm or 11.5 mm long implants: 5-year results from a randomised controlled trial. *Eur. J. Oral Implantol.* **2018**, *11*, 295–306.
38. Storelli, S.; Abbà, A.; Scanferla, M.; Botticelli, D.; Romeo, E. 6 mm vs 10 mm-long implants in the rehabilitation of posterior jaws: A 10-year follow-up of a randomised controlled trial. *Eur. J. Oral Implantol.* **2018**, *11*, 283–292.
39. Naenni, N.; Sahrmann, P.; Schmidlin, P.R.; Attin, T.; Wiedemeier, D.B.; Sapata, V.; Hämmerle, C.H.F.; Jung, R.E. Five-Year Survival of Short Single-Tooth Implants (6 mm): A Randomized Controlled Clinical Trial. *J. Dent. Res.* **2018**, *97*, 887–892.
40. Meloni, S.M.; Lumbau, A.; Spano, G.; Baldoni, E.; Pisano, M.; Tullio, A.; Tallarico, M. Sinus augmentation grafting with anorganic bovine bone versus 50% autologous bone mixed with 50% anorganic bovine bone: 5 years after loading results from a randomised controlled trial. *Int. J. oral Implantol. (Berlin, Ger.* **2019**, *12*, 483–492.
41. Testori, T.; Panda, S.; Clauser, T.; Scaini, R.; Zuffetti, F.; Capelli, M.; Taschieri, S.; Mortellaro, C.; Del Fabbro, M. Short implants and platelet-rich fibrin for transcrestal sinus floor elevation: a prospective multicenter clinical study. *J. Biol. Regul. Homeost. Agents* **2019**, *33*, 121-135. DENTAL SUPPLEMENT.
42. Slot, W.; Raghoebar, G.M.; Cune, M.S.; Vissink, A.; Meijer, H.J.A. Four or six implants in the maxillary posterior region to support an overdenture: 5-year results from a randomized controlled trial. *Clin. Oral Implants Res.* **2019**, *30*, 169–177.
43. Qian, S.-J.; Mo, J.-J.; Si, M.-S.; Qiao, S.-C.; Shi, J.-Y.; Lai, H.-C. Long-term outcomes of osteotome sinus floor elevation with or without bone grafting: The 10-year results of a randomized controlled trial. *J. Clin. Periodontol.* **2020**, *47*, 1016–1025.
44. Gulјé, F.L.; Meijer, H.J.A.; Abrahamsson, I.; Barwacz, C.A.; Chen, S.; Palmer, P.J.; Zadeh, H.; Stanford, C.M. Comparison of 6-mm and 11-mm dental implants in the posterior region supporting fixed dental prostheses: 5-year results of an open multicenter randomized controlled trial. *Clin. Oral Implants Res.* **2021**, *32*, 15–22.
45. Zadeh, H.H.; Guljé, F.; Palmer, P.J.; Abrahamsson, I.; Chen, S.; Mahallati, R.; Stanford, C.M. Marginal bone level and survival of short and standard-length implants after 3 years: An Open Multi-Center Randomized Controlled Clinical Trial. *Clin. Oral Implants Res.* **2018**, *29*, 894–906.
46. Barausse, C.; Felice, P.; Pistilli, R.; Buti, J.; Rcsed, M.; Esposito, M. Posterior Jaw Rehabilitation Using Partial Prostheses Supported By Implants 4.0 X 4.0 Mm or Longer: Three-Year Post-Loading Results of a Multicentre Randomised Controlled Trial. *Clin. Trials Dent.* **2019**, *1*, 25–36.
